# Evaluating plantar biomechanics while descending a single step with different heights

**DOI:** 10.3389/fbioe.2024.1431988

**Published:** 2024-08-12

**Authors:** Panjing Guo, Xiajing Zhang, Haoran Xu, Ruiqin Wang, Yumin Li, Chengshuo Xu, Yu Yang, Linlin Zhang, Roger Adams, Jia Han, Jie Lyu

**Affiliations:** ^1^ Department of Orthopedics, Jinshan District Central Hospital Affiliated to Shanghai University of Medicine and Health Sciences, Shanghai, China; ^2^ College of Rehabilitation Sciences, Shanghai University of Medicine and Health Sciences, Shanghai, China; ^3^ College of Medical Instruments, Shanghai University of Medicine and Health Sciences, Shanghai, China; ^4^ Research Institute for Sport and Exercise, Faculty of Health, University of Canberra, Canberra, Australia

**Keywords:** single transition step descent, plantar pressure, plantar pressure center parameters, plantar pressure distribution parameters, landing strategy, dynamic balance

## Abstract

**Objective:**

This study aims to investigate the plantar biomechanics of healthy young males as they descend a single transition step from varying heights.

**Methods:**

Thirty healthy young males participated the experiment using the F-scan insole plantar pressure system in which participants made single transition steps descent from four step heights (5, 15, 25, and 35 cm), leading with their dominant or non-dominant foot. Plantar pressure data were collected for 5 s during the period between landing touchdown and standing on the ground. Landing at each step height was repeated three times, with a five-minute rest between different height trials.

**Results:**

At 5 cm and 15 cm steps, participants demonstrated a rearfoot landing strategy on both sides. However, forefoot contact was observed at heights of 25 cm and 35 cm. Parameters related to center of plantar pressure (COP) of the leading foot were significantly larger compared to the trailing foot (*P* < 0.001), increased with higher step heights. Vertical ground reaction forces for the biped, leading and trailing feet decreased with increasing step height (all *P* < 0.05). The leading foot had a higher proportion of overall and forefoot loads, and a lower proportion of rearfoot load compared to the trailing foot (*P* < 0.001). The overall load on the dominant side was lower than that on the non-dominant side for both the leading and trailing feet (*P* < 0.001). For the trailing foot, forefoot load on the dominant side was lower than that on the non-dominant side, however, the opposite result appeared in rearfoot load (*P* < 0.001). Upon the leading foot landing, forefoot load exceeded the rearfoot load for the dominant (*P* < 0.001) and non-dominant sides (*P* < 0.001). Upon the trailing foot landing, forefoot load was lower than the rearfoot load for the dominant (*P* < 0.001) and non-dominant sides (*P* = 0.019).

**Conclusion:**

When the characteristics of biomechanical stability are compromised by step height, landing foot, and footedness factors — due to altered foot landing strategies, changing COP, or uneven force distribution — ability to control motion efficiently and respond adaptively to the forces experienced during movement is challenged, increasing the likelihood of loss of dynamic balance, with a consequent increased risk of ankle sprains and falls.

## 1 Introduction

Ankle sprain, a common musculoskeletal injury, typically occurs during activities such as jumping, landing, and stair descent, which all involve foot inversion and may result in falls (Kang et al., 2022). However, the specific biomechanical demands differ. Jumping emphasizes impact absorption and rapid stabilization, and multi-step stair descent involves continuous muscle activity and greater joint moments. Descending a single transition step—a frequent daily activity—is a complex task that imposes heightened demands on the skeletal, neural, and muscular systems of the lower limbs, focusing on controlled, balanced movement and controlled eccentric contractions (Protopapadaki et al., 2007). The transition area between the step and level ground (Sheehan and Gottschall, 2011) is frequently implicated in lower extremity injuries such as ankle sprains (Peng et al., 2016). Specifically, during the transition from step to flat ground, the process commences with the movement of one foot from the edge of the step to the ground. Subsequently, the other foot follows, stepping off to join the first (Alcock et al., 2014), providing necessary support for one limb and facilitating the next step for the other limb. Errors in this sequence can lead to injuries of the foot and ankle complex, making stepping down from a single transition step risky. Approximately 23% of fall-related lower extremity injuries occur on curbs or steps, with 30% of these step-related falls happening during the first or last step of the transition to level ground (Koepsell et al., 2004). This sequence poses a challenging and high-risk task for individuals in community settings (Templer, 1995). However, limited research is available on the mechanisms of descending a single transition step (Begg and Sparrow, 2000; Lythgo et al., 2007; van Dieën and Pijnappels, 2009). A kinematic study comparing multi-step descents to transitions to level ground revealed greater variability in lower limb kinematics during the transition step (Yu et al., 1997), suggesting that this variability could increase the likelihood of missteps or falls. Consequently, the theoretical framework in relation to continuous descent may not be directly applicable to a single transition step descent. Consequently, it is of great importance to investigate plantar biomechanics during the sequence of events in a single step descent to understand its correlation with lower extremity injuries, which constitute significant clinical and societal public health concerns (Yu et al., 1997; Sheehan and Gottschall, 2011).

Numerous factors influence movement control when descending a single transition step, with step height (Gerstle et al., 2017; Guo et al., 2023), landing foot (Gerstle et al., 2021), and footedness (Wang and Fu, 2019) identified as key variables. Observations from daily life indicate that, the higher the step, the greater the demands on lower limb neuromuscular control and dynamic balance stabilization. However, current research is insufficient regarding the effects of step height variations on landing strategies, postural control, and balance. Existing research indicates that as step height increases from 0 cm (100% rearfoot strike) to 20 cm (63.6% forefoot strike), the prevalence of forefoot landing strategies increases (Freedman and Kent, 1987). Yet, at a height difference of only 5 cm, forefoot use for step descent is almost unobservable (van der Linden et al., 2007). As step height increases, controlling forward momentum becomes crucial, and forefoot landing is more consistently employed (Riener et al., 2002; Spanjaard et al., 2009). Currently, it is unclear how the shift between forefoot and rearfoot landing strategies occurs at various step heights, and how this influences dynamic balance. One previous study examined the asymmetry between dominant and nondominant legs in lower limb biomechanics (Wang and Fu, 2019), suggesting distinct biomechanical characteristics in different landing feet. Thus, understanding how step height, landing foot choice, and individual footedness influence control during single transition step descent is crucial due to its significant ergonomic implications. These results could help reduce the risk of fall-related ankle injuries, enhance human convenience, and optimize the living environment. Accordingly, this study aims to explore foot landing strategies employed during the initial contact and weight acceptance phases of descending a single transition step. Based on prior studies (Freedman and Kent, 1987; Gerstle et al., 2017), it is hypothesized that at lower step heights, participants will predominantly make initial contact with their rearfoot, gradually shifting to forefoot as step height increases.

Plantar pressure is a critical component of standing and walking (Gao et al., 2022). Plantar pressure detecting and analyzing can increase awareness of potential hazards for fall-related lower extremity injuries (Niu et al., 2019). Currently, only three studies have examined how step-down techniques (rearfoot vs. forefoot) influence plantar pressure when performing a curb descent task. van Dieën et al. (2008) observed that individuals exhibited lower vertical ground reaction forces (vGRF) when adopting a forefoot technique compared to a rearfoot technique. In contrast, Moudy et al. (2020) found no differences in vGRF between individuals who naturally used a forefoot technique and those who used a rearfoot technique. However, Demers et al. (2021) found that vGRF were higher when subjects employed the forefoot technique. Given these inconsistent findings regarding the vGRF on the rearfoot and forefoot when contacting the ground, further investigation is warranted. Consequently, our study employed a plantar pressure testing device to investigate the biomechanical characteristics of the plantar surface during the descent of a single transition step from progressively increasing heights, using alternating landing feet.

## 2 Materials and methods

### 2.1 Participants

The inclusion criteria were: ① Ages between 18 and 30 years; ② No history of related injuries or diseases affecting postural and balance control within the past 6 months, including foot and ankle injuries, neurological diseases, lower limb fractures, leg length discrepancies, or arthritis; ③ No ongoing use of medications that affect balance function; ④ Completion of a questionnaire and provision of signed informed consent.

Given the diminished motor function in older adults and the associated risk of injury, healthy young males were recruited. Accordingly, thirty healthy males with a mean age of 23.9 ± 1.2 years, height of 176.9 ± 6.1 cm, weight of 76.0 ± 11.9 kg, and shoe sizes ranging from 41 to 43 Euro Size participated in this study. All were right-footed, as determined by the Chinese version of the Waterloo Footedness Questionnaire (Yang et al., 2018). The experimental protocol was approved by the Human Research Ethics Committee of Shanghai University of Sport (approval number: 102772021RT073). All experiments were performed in accordance with the Declaration of Helsinki. Informed consent was obtained prior to participation.

### 2.2 Procedures

#### 2.2.1 Pre-test preparation

**FIGURE 1:**
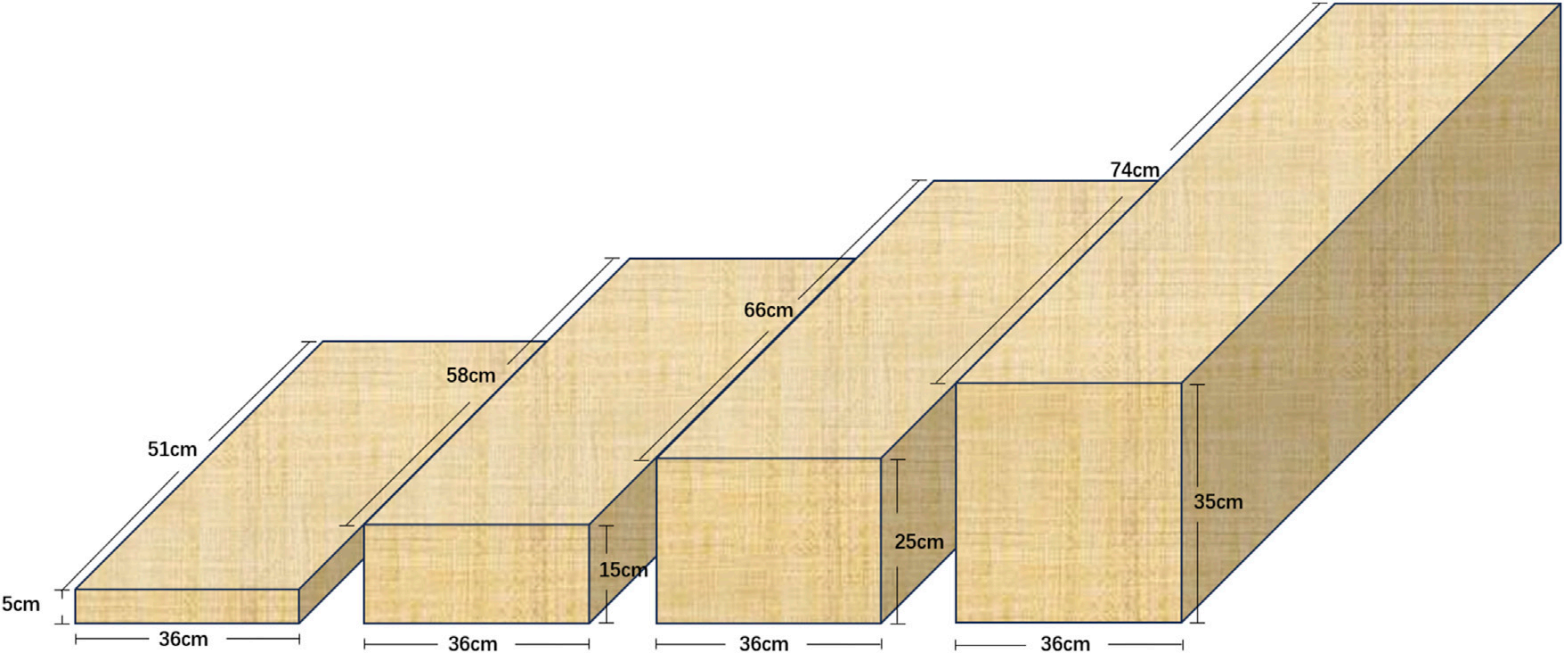
Steps at heights of 5, 15, 25, and 35 cm.

The experiment was conducted in a quiet room to minimize external disturbances. Four wooden steps with heights of 5 cm, 15 cm, 25 cm, and 35 cm were used, with respective dimensions of 51 × 36 × 5 cm, 58 × 36 × 15 cm, 66 × 36 × 25 cm, and 74 × 36 × 35 cm, as illustrated in Figure 1. The heights of 5 cm, 15 cm, and 25 cm correspond to standard curb and building code step heights, and are also 2.5 cm higher than the current guidelines of the United States Federal Highway Administration (Gerstle et al., 2017). The 35 cm step was included to simulate a larger and more challenging daily activity step.

**FIGURE 2:**
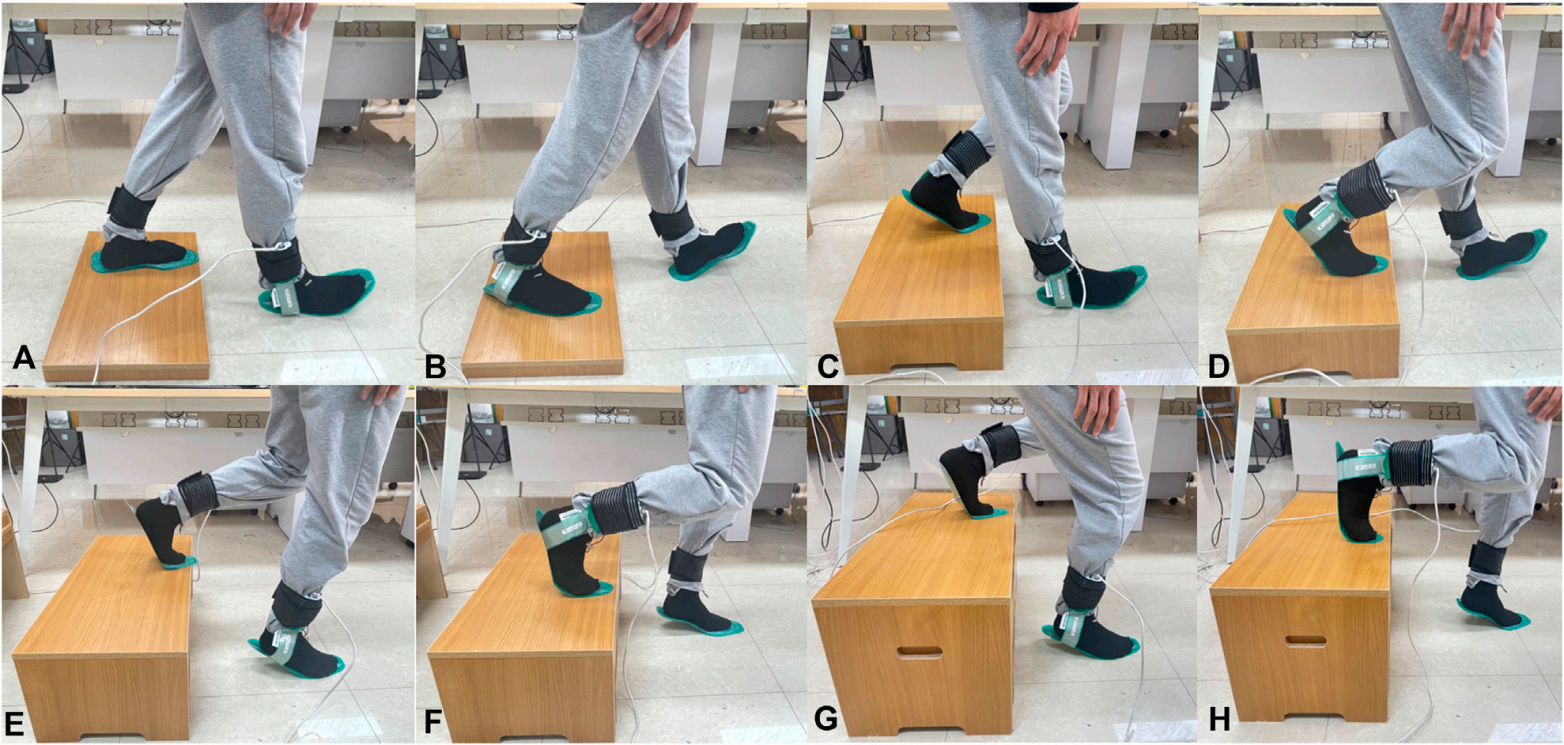
The transitional step descent experiments from different step heights. **(A)** Descending from a 5 cm step with the right foot as the leading foot; **(B)** Descending from a 5 cm step with the left foot as the leading foot; **(C)** Descending from a 15 cm step with the right foot as the leading foot; **(D)** Descending from a 15 cm step with the left foot as the leading foot; **(E)** Descending from a 25 cm step with the right foot as the leading foot; **(F)** Descending from a 25 cm step with the left foot as the leading foot; **(G)** Descending from a 35 cm step with the right foot as the leading foot; **(H)** Descending from a 35 cm step with the left foot as the leading foot.

Participants conducted single transition step experiments descending from steps of four different heights (5, 15, 25, and 35 cm), using both the right and left foot as the leading foot in a randomized sequence. Each condition was tested to include both feet as the leading foot, as detailed in Figure 2.

The insole system used was an F-scan plantar pressure analysis system (Tekscan, Boston, MA, United States), providing real-time monitoring and feedback of the “foot-shoe interface” pressure throughout the entire support phase. Actually, this device is favoured for its flexibility, mobility, simplicity and suitability for a wide range of media with different materials and characteristics. The advantage is that the subject can use a natural gait during the experiment, avoiding problems such as platform aiming (Ledoux et al., 2013). Therefore, due to its portability in shoes or socks, the device is suitable for daily habitual or wider range of sporting activities, indoors or outdoors (Mei et al., 2015). However, as postural control appears to be related to plantar sensitivity, a limitation of the system is that the sensitivity of the sensor performance may be disturbed when insoles are inserted in the shoe (Machado et al., 2017). In addition, the insole has a limited number of sensors that only cover the area inside the shoe, which is not as comprehensive as a force plate or force table system (Putti et al., 2007). Besides, the performance of insole-based sensors decreases through multiple experiments and increasing experiment time. Finally, heat and sweat from the foot inside the shoe can also affect the in-shoe sensors, which may lead to biased results (Woodburn and Helliwell, 1996). All participants used the same type of size-adjustable testing insole, with a thickness of 0.15 mm. Each insole had four piezoresistive sensors per 1 cm^2^, with a measurement range of 0 kPa to 862 kPa. The sampling frequency was set at 50 Hz. Due to the softness of the shoe lining material, inserting the force-measuring insoles into the shoes may cause wrinkles that could affect data accuracy. To address this issue, participants removed their shoes during the test and wore uniform cotton socks. These socks served as the medium to securely adhere the force-measuring insole to the subjects’ toes, arches, and heels using regular double-sided adhesive. This approach prevented shifts in the relative positions of the test insole and the subjects’ feet during preparation and the standing process, ensuring uniform data measurement positions.

#### 2.2.2 Testing procedure

①Single transition step descent: Participants engaged in an exercise involving walking down a single step, during which they were instructed to move their pelvises forward and backward (anterior-posterior) and side to side (medial-lateral) to maintain an even pressure distribution across the soles of their feet. Participants were instructed to keep their eyes level, gaze straight ahead, and maintain a relaxed, natural posture while descending. When completing the step and standing flat on the ground, plantar pressure data were collected for 5 s. This part of the experiment was repeated three times for each step height, with a five-minute rest period between trials.

②Strategy Assessment: Participants completed each step height without specific landing strategy guidance until three consistent landings (either rearfoot or forefoot) were recorded (individual preferred landing strategy), establishing the participant’s landing strategy to minimize variability. Throughout the experiment, a second experimenter observed each participant’s landing from the side to visually assess the landing strategy. A rearfoot landing strategy involved initial contact with the heel, characterized by dorsiflexion (direction of toe force upward), during weight acceptance. Conversely, a forefoot landing strategy entailed landing in a neutral position or with forefoot contact, characterized by toe flexion (direction of toe force downward), during weight acceptance (Gerstle et al., 2017).

③Safety Measures: A third experimenter positioned themselves behind the participants to prevent falls throughout the experiment.

#### 2.2.3 Data processing

Plantar pressure data, including raw data of the center of plantar pressure (COP) and distribution for each frame, were exported from the F-scan plantar pressure analysis system. COP oscillation is widely recognized as a key parameter in assessing postural stability (Pinsault and Vuillerme, 2009; Paillard and Noé, 2015). Customized Python programs (PyCharm Community Edition 2022.2, JetBrains s. r.o., Prague, Czech Republic) were used for data processing and exporting relevant parameters, categorized into two groups. Plantar pressure parameters were defined as outlined in Table 1 (Guo et al., 2023). Plantar pressure center parameters included COP-ML adjustment velocity (mm/s), COP-AP adjustment velocity (mm/s), COP adjustment velocity (mm/s), 95% confidence circle area (mm^2^), ML range (mm), AP range (mm), maximum swing (mm), minimum swing (mm), mean X (mm), and mean Y (mm). Plantar pressure distribution parameters included ground reaction forces for the biped, leading and trailing feet, as well as overall, forefoot, and rearfoot loads. Finally, COP localization was respectively determined by mean X and Y coordinates along the *X*-axis and *Y*-axis.

**TABLE 1:** Formulas related to kinematic parameters.

Kinematic parameters	Formula
COP Total adjustment time (s)	COP Total adjustment time=T
COP-ML adjustment velocity (mm/s)	$COP-ML\;adjustment\;velocity=1/T\sum_{n=1}^{N-1}\left|ML\right.\left[n+1\right]\left.-ML\left[n\right]\right|$
COP-AP adjustment velocity (mm/s)	$COP-AP\;adjustment\;velocity=1/T\sum_{n=1}^{N-1}\left|AP\right.\left[n+1\right]\left.-AP\left[n\right]\right|$
COP adjustment velocity (mm/s)	$COP\;adjustment\;velocity=1/T\sum_{n=1}^{N-1}{\left[{\left(AP\left[n+1\right]-AP\left[n\right]\right)}^{2}+{\left(ML\left[n+1\right]-ML\left[n\right]\right)}^{2}\right]}^{1/2}$
95% confidence circle area (mm^2^)	$Mean\;Distance=1/N\sum_{n=1}^{N}{\left[AP{\left[n\right]}^{2}+ML{\left[n\right]}^{2}\right]}^{1/2}$
	$RMS\;Distance={\left[1/N\sum_{n=1}^{N}\left[AP{\left[n\right]}^{2}+ML{\left[n\right]}^{2}\right]\right]}^{1/2}$
	$95\%\;confidence\;circle\;area=\pi {\left(MDIST+1.645{\left[{RDIST}^{2}-{MDIST}^{2}\right]}^{1/2}\right)}^{2}$
ML range (mm)	$ML\;range=\max_{1\leq n\leq m\leq N}|\left.ML\left[n\right]-ML\left[m\right]\right|$
AP range (mm)	$AP\;range=\max_{1\leq n\leq m\leq N}|\left.AP\left[n\right]-AP\left[m\right]\right|$
Maximum swing (mm)	${Maximum\;swing=\max}_{1\leq n\leq N-1}{\left[{\left(AP\left[n+1\right]-AP\left[n\right]\right)}^{2}+{\left(ML\left[n+1\right]-ML\left[n\right]\right)}^{2}\right]}^{1/2}$
Minimum swing (mm)	$Minimum\;swing=\min_{1\leq n\leq N-1}{\left[{\left(AP\left[n+1\right]-AP\left[n\right]\right)}^{2}+{\left(ML\left[n+1\right]-ML\left[n\right]\right)}^{2}\right]}^{1/2}$
Mean X (mm)	$Mean\;X=\frac{1}{N}\sum_{n=1}^{N}{ML}_{n}$
Mean Y (mm)	$Mean\;Y=\frac{1}{N}\sum_{n=1}^{N}{AP}_{n}$

### 2.3 Statistical analysis

Data were analyzed with SPSS Statistics (version 26.0; IBM, Chicago, IL, United States) and Excel 2016 (Microsoft, Chagrin Falls, OH, United States), and scatter plots were created using GraphPad Prism 9 (GraphPad Software, San Diego, CA, United States). Chi-square tests were used to analyze the relationship between different step heights and landing strategies, and between footedness and landing strategies. For plantar pressure center parameters, a three-way analysis of variance (ANOVA) was initially performed to identify significant factors. Subsequently, a two-way ANOVA with repeated measures was conducted to examine the main effects and interactions between pairs of factors, with the Bonferroni correction applied for post hoc multiple comparisons. For vGRF, a one-way ANOVA was used to assess differences across the four step heights, with Bonferroni correction applied for post hoc multiple comparisons. For plantar pressure distribution parameters, a paired t-test was used for normally distributed data, and the Wilcoxon rank sum test was applied to skewed distributions. Normally distributed data are expressed as mean ± standard deviation (M ± SD). The significance level α was set a priori at 0.05.

## 3 Results

### 3.1 Foot landing strategy

For both the dominant and non-dominant sides as the leading foot, the majority of participants initially favored a rearfoot landing strategy at lower step heights, at 93.33% (28/30) and 90% (27/30) respectively at 5 cm, and 56.67% (17/30) and 53.33% (16/30) respectively at 15 cm. However, as the step height increased, a shift towards a forefoot landing strategy was observed. At 25 cm, 80% (24/30) on the dominant side and 86.67% (26/30) on the non-dominant side preferred the forefoot strategy. This trend was further pronounced at 35 cm, where 96.67% (29/30) of participants on both sides opted for a forefoot landing strategy.

**FIGURE 3:**
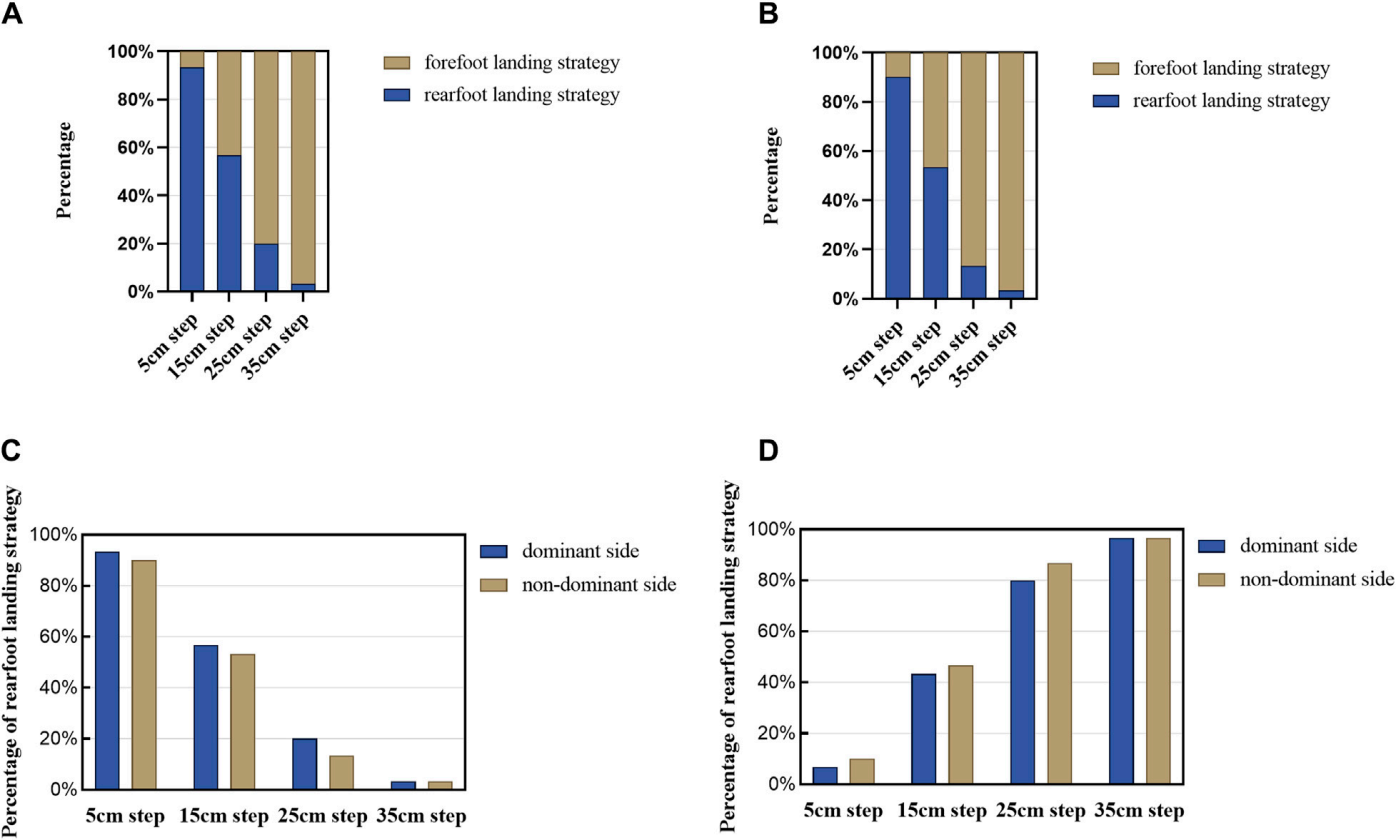
Foot landing strategy. **(A)** Foot landing strategy on the dominant side. **(B)** Foot landing strategy on the non-dominant side. **(C)** Rearfoot landing strategy of dominant vs. non-dominant side. **(D)** Forefoot landing strategy of dominant vs. non-dominant side.

The results of the chi-square test showed a significant difference in landing strategies between step heights (dominant side: *X* ^2^ = 58.91, *P* < 0.001; non-dominant side: *X* ^2^ = 59.17, *P* < 0.001). There was no significant difference in landing strategies between dominant and non-dominant sides (*X* ^2^ = 0.274, *P* = 0.6). The foot landing strategies for both dominant and non-dominant sides are illustrated in Figure 3.

### 3.2 Parameters related to center of plantar pressure

#### 3.2.1 Parameters related to the center of plantar pressure for different feet at different step heights

The analysis of COP parameters through a three-way ANOVA, which considered factors of step height, landing foot, and footedness, revealed no statistically significant differences between the dominant and non-dominant sides. Additionally, further exploration using a two-way ANOVA with repeated measures assessed the impact between step height and descending foot. The results, detailed in Table 2, showed no significant interactions.

**TABLE 2:** Comparison of COP data on transition steps for different feet at different step heights.

Items	5 cm step		15 cm step		25 cm step		35 cm step	
	Leading foot	Trailing foot	Leading foot	Trailing foot	Leading foot	Trailing foot	Leading foot	Trailing foot
Dominant side								
COP-ML adjustment velocity (mm/s)	11.10 ± 4.39	7.01 ± 2.43	13.77 ± 5.13	9.79 ± 4.81	13.76 ± 5.20	10.30 ± 4.48	15.35 ± 4.33	11.45 ± 3.98^a^ ^,^ ^b^
COP-AP adjustment velocity (mm/s)	55.70 ± 21.53	40.33 ± 13.92	63.92 ± 24.58	51.51 ± 26.52	63.69 ± 17.82	51.20 ± 19.97	75.99 ± 23.51	54.36 ± 21.39^a^ ^,^ ^b^
COP adjustment velocity (mm/s)	58.55 ± 21.94	42.14 ± 14.28	67.55 ± 25.19	53.87 ± 26.94	67.62 ± 18.94	53.77 ± 20.53	80.06 ± 23.32	57.70 ± 21.87^a^ ^,^ ^b^
95% confidence circle area (mm^2^)	3403.41 ± 2036.14	2771.56 ± 2553.56	4495.04 ± 2814.83	2705.70 ± 2277.62	5284.63 ± 3880.05	3269.67 ± 2545.72	7236.10 ± 4644.70	3052.39 ± 2506.51^a^ ^,^ ^b^
ML range (mm)	20.14 ± 12.47	9.26 ± 3.02	26.44 ± 13.04	16.04 ± 9.67	23.50 ± 12.04	15.87 ± 10.31	23.60 ± 9.78	16.60 ± 9.16^a^ ^,^ ^b^
AP range (mm)	109.70 ± 23.61	92.58 ± 24.92	118.52 ± 23.51	98.49 ± 32.47	120.45 ± 29.02	102.74 ± 24.35	133.50 ± 27.73	108.10 ± 29.62^a^ ^,^ ^b^
Maximum swing (mm)	45.83 ± 34.57	35.96 ± 26.93	43.58 ± 24.94	54.13 ± 42.63	40.49 ± 22.27	56.83 ± 39.39	52.50 ± 25.70	62.81 ± 34.98^a^
Minimum swing (mm)	0.01 ± 0.01	0.01 ± 0.01	0.01 ± 0.01	0.01 ± 0.01	0.01 ± 0.01	0.01 ± 0.01	0.01 ± 0.01	0.01 ± 0.01
Mean X (mm)	54.51 ± 4.05	54.05 ± 4.60	54.54 ± 4.03	54.27 ± 4.22	55.11 ± 5.70	56.02 ± 6.64	55.74 ± 5.99	56.56 ± 6.35
Mean Y (mm)	141.78 ± 22.30	127.63 ± 18.67	145.67 ± 22.70	125.28 ± 19.49	144.19 ± 26.85	120.35 ± 23.97	146.78 ± 24.59	119.62 ± 25.95^b^
Non-dominant side								
COP-ML adjustment velocity (mm/s)	9.47 ± 2.74	8.08 ± 3.09	12.14 ± 3.95	9.87 ± 4.38	13.38 ± 3.45	10.05 ± 4.08	15.87 ± 3.92	11.13 ± 4.80^a^ ^,^ ^b^
COP-AP adjustment velocity (mm/s)	50.22 ± 9.53	42.20 ± 16.43	56.68 ± 23.11	48.41 ± 21.70	64.27 ± 17.89	53.20 ± 22.87	76.26 ± 22.25	52.72 ± 22.05^a^ ^,^ ^b^
COP adjustment velocity (mm/s)	52.61 ± 9.56	44.67 ± 16.54	59.98 ± 23.17	50.84 ± 22.17	68.11 ± 17.31	55.76 ± 23.18	80.38 ± 22.36	55.67 ± 22.40^a^ ^,^ ^b^
95% confidence circle area (mm^2^)	2893.49 ± 1656.56	1944.36 ± 1510.72	4265.14 ± 2058.55	2797.32 ± 2818.99	4950.06 ± 2919.33	3260.88 ± 2939.53	6005.57 ± 3120.30	3312.46 ± 2436.16^a^ ^,^ ^b^
ML range (mm)	16.11 ± 5.92	16.36 ± 10.47	22.98 ± 11.56	17.22 ± 9.51	23.40 ± 10.87	17.48 ± 9.01	26.86 ± 10.93	19.26 ± 11.63^a^ ^,^ ^b^
AP range (mm)	98.80 ± 18.93	90.71 ± 27.84	120.81 ± 24.70	97.33 ± 32.66	120.18 ± 28.30	101.66 ± 33.13	140.71 ± 29.10	104.04 ± 32.60^a^ ^,^ ^b^
Maximum swing (mm)	45.20 ± 25.22	48.34 ± 38.59	43.45 ± 21.56	52.88 ± 44.97	45.58 ± 17.30	59.76 ± 39.17	52.83 ± 30.45	54.83 ± 41.65
Minimum swing (mm)	0.01 ± 0.01	0.01 ± 0.01	0.01 ± 0.01	0.01 ± 0.01	0.01 ± 0.01	0.01 ± 0.01	0.01 ± 0.01	0.01 ± 0.01
Mean X (mm)	52.20 ± 3.97	51.70 ± 3.76	50.64 ± 5.01	51.47 ± 5.21	49.80 ± 4.54	49.98 ± 4.40	48.45 ± 4.56	49.67 ± 4.20^a^
Mean Y (mm)	142.90 ± 21.02	133.85 ± 23.00	138.37 ± 19.68	127.74 ± 21.50	140.52 ± 23.53	124.43 ± 22.57	131.07 ± 23.51	125.96 ± 26.56^b^

^a^ Indicates statistically significant differences between different step heights of 5, 15, 25, and 35 cm at *P* < 0.05;

^b^ Indicates statistically significant differences between different feet with the leading foot and trailing foot at *P* < 0.05.

In Table 2, comparing the four step heights of 5, 15, 25, and 35 cm, the 95% confidence circle area (mm^2^) (*P* = 0.002), ML range (mm) (*P* = 0.003), AP range (mm) (*P* = 0.002), Maximum swing (mm) (*P* = 0.046) on the dominant side and ML range (mm) (*P* = 0.002), Mean X (mm) (*P* = 0.003) on the non-dominant side are statistically different. The differences in COP-ML adjustment velocity (mm/s), COP-AP adjustment velocity (mm/s), COP adjustment velocity (mm/s) on the dominant side and COP-ML adjustment velocity (mm/s), COP-AP adjustment velocity (mm/s), COP adjustment velocity (mm/s), 95% confidence circle area (mm^2^), and AP range (mm) on the non-dominant side are statistically significant (*P* < 0.001). All the above parameters increase with the increase in height. When comparing the leading foot and trailing foot, the differences in COP-ML adjustment velocity (mm/s), COP-AP adjustment velocity (mm/s), COP adjustment velocity (mm/s), 95% confidence circle area (mm^2^), ML range (mm), AP range (mm), and Mean Y (mm) are all statistically significant (*P* < 0.001) for both the dominant and non-dominant sides. Consequently, these parameters are consistently higher when the leading foot lands compared to the trailing foot during the single transition step descent.

#### 3.2.2 Comparison of plantar pressure center scatter plots

**FIGURE 4:**
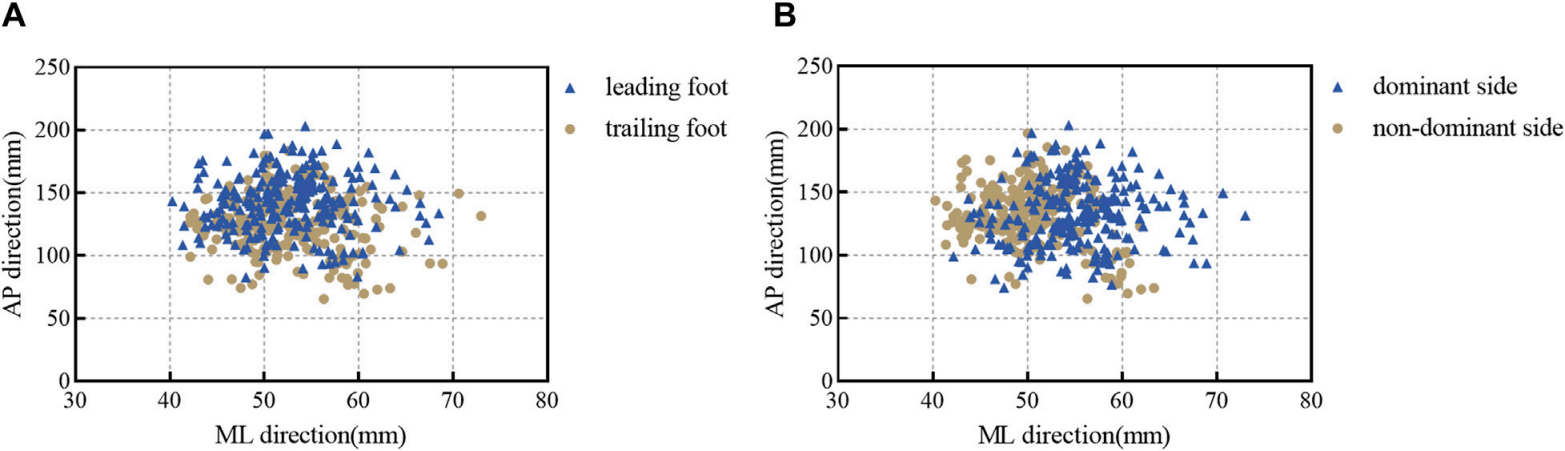
Scatter diagram of COP. **(A)** Comparison of the COP in the leading and trailing feet. **(B)** Comparison of the COP in the dominant and non-dominant sides.

Examination of the scatter plots for mean X and mean Y values for the leading and trailing feet reveals a significant difference in the anterior-posterior (AP) direction. Specifically, the mean Y value of the leading foot is significantly greater than that of the trailing foot, as illustrated in Figure 4A. Similarly, scatter plots for mean X and mean Y values of the dominant and non-dominant sides show a significant difference in the medial-lateral (ML) direction, with the mean X of the dominant side significantly greater than that of the non-dominant side, as depicted in Figure 4B.

### 3.3 Parameters related to plantar pressure distribution

#### 3.3.1 Relationship between vertical ground reaction forces and different step heights for the biped, leading and trailing feet

**FIGURE 5:**
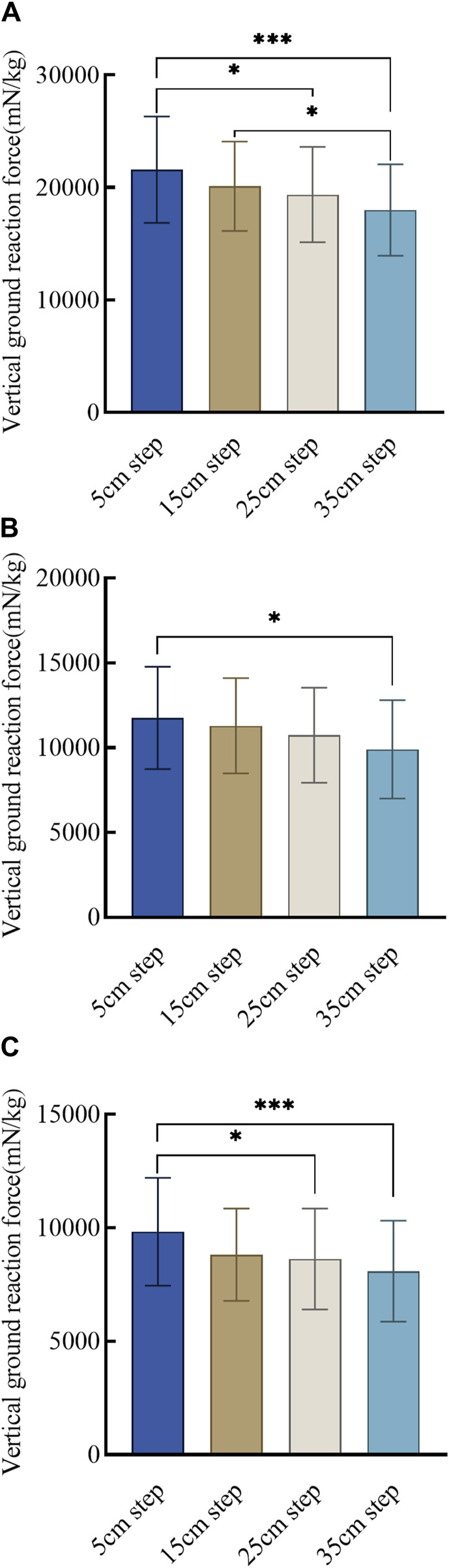
Vertical ground reaction force values for transition steps at varying step heights. **(A)** Vertical ground reaction bipedal force at different step heights for transition step; **(B)** Vertical ground reaction force of the leading foot at different step heights for transition step; **(C)** Vertical ground reaction force of the trailing foot at different step heights for transition step. Note: * indicates statistically significant difference at *P* < 0.05, and *** indicates statistically significant difference at *P* < 0.001.

ANOVA results indicated statistically significant differences in vGRF for the biped across the four step heights (*P* < 0.001). Post-hoc multiple comparisons using the Bonferroni correction reveal specific differences: 5 cm step vs. 15 cm step (*P* = 0.349), 5 cm step vs. 25 cm step (*P* = 0.028), 5 cm step vs. 35 cm step (*P* < 0.001), 15 cm step vs. 25 cm step (*P* = 0.999), 15 cm step vs. 35 cm step (*P* = 0.043), and 25 cm step vs. 35 cm step (*P* = 0.475). Detailed data are provided in Supplementary Tables S1, S2. These post-hoc analysis results are illustrated in Figure 5A.

For the leading foot, vGRF shows significant differences across the four step heights (*P* = 0.004). Post-hoc comparisons with Bonferroni correction show specific differences: 5 cm step vs. 15 cm step (*P* = 0.999), 5 cm step vs. 25 cm step (*P* = 0.321), 5 cm step vs. 35 cm step (*P* = 0.003), 15 cm step vs. 25 cm step (*P* = 0.999), 15 cm step vs. 35 cm step (*P* = 0.054), and 25 cm step vs. 35 cm step (*P* = 0.690). Detailed data are provided in Supplementary Tables S1, S2. The post-hoc analysis results are presented in Figure 5B.

For the trailing foot, vGRF demonstrates significant differences across the four step heights (*P* < 0.001). Post-hoc comparisons with Bonferroni correction show specific differences: 5 cm step vs. 15 cm step (*P* = 0.078), 5 cm step vs. 25 cm step (*P* = 0.020), 5 cm step vs. 35 cm step (*P* < 0.001), 15 cm step vs. 25 cm step (*P* = 0.999), 15 cm step vs. 35 cm step (*P* = 0.454), and 25 cm step vs. 35 cm step (*P* = 0.999). Detailed data are provided in Supplementary Tables S1, S2. The post-hoc analysis results are depicted in Figure 5C.

Figure 5 clearly shows significant differences in the post-hoc comparisons of vGRF for the biped when the step height difference is 20 cm or more. Specifically, a significant difference in vGRF for the leading foot is observed in the post-hoc comparison when the step height difference is 30 cm. For the trailing foot, significant differences in the post-hoc comparisons of vGRF are evident only when the step height difference is 20 cm or more.

#### 3.3.2 Parameters of plantar pressure distribution on the dominant and non-dominant sides when they are the leading foot and the trailing foot

According to Table 3, regardless of whether the dominant or non-dominant side acts as the leading foot, the leading foot bears a higher proportion of overall load compared to the trailing foot (*P* < 0.001). When the dominant side acts as the leading foot and the non-dominant side acts as the trailing foot, the difference between the two is less than when the non-dominant side acts as the leading foot and the dominant side acts as the trailing foot. When the dominant side is used as the leading foot or the trailing foot, the proportion of forefoot load has a significant difference (*P* < 0.001); When the non-dominant side is used as the leading foot and trailing foot, the proportion of forefoot load has a significant difference (*P* < 0.001), and the forefoot load of the leading foot is significantly greater than that of the trailing foot. When the dominant side is used as the leading foot or the trailing foot, the proportion of rearfoot load has a significant difference (*P* < 0.001); When the non-dominant side is used as the leading foot or the trailing foot, the proportion of rearfoot load has a significant difference (*P* < 0.001), and the rearfoot load of the leading foot is significantly lower than that of the trailing foot.

**TABLE 3:** Plantar pressure distribution data when the dominant and non-dominant sides act as the leading and trailing feet.

Items	Location	Leading foot (M ± SD, %)	Trailing foot (M ± SD, %)	t	*p*
Overall load	Dominant side as leading foot; Non-dominant side as trailing foot	51.96 ± 6.20	48.04 ± 6.20	3.46	<0.001
	Non-dominant side as leading foot; Dominant side as trailing foot	58.16 ± 6.18	41.84 ± 6.18	14.48	<0.001
Forefoot load	Dominant side	55.54 ± 16.42	40.45 ± 15. 96	8.80	<0.001
	Non-dominant side	54.69 ± 14.63	46.64 ± 15.47	4.89	<0.001
Rearfoot load	Dominant side	44.46 ± 16.42	59.55 ± 15.96	−8.80	<0.001
	Non-dominant side	45.31 ± 14.63	53.36 ± 15.47	−4.89	<0.001

#### 3.3.3 Comparison of plantar pressure distribution parameters between the dominant and non-dominant sides

As shown in Table 4, there is a significant difference in overall load between the dominant and non-dominant sides (*P* < 0.001), with the dominant side accounting for a lower percentage of the overall load than the non-dominant side when descending as a leading foot, and the dominant side accounting for a similarly lower percentage of the overall load than the non-dominant side when descending as a trailing foot. Regarding forefoot load, there is no significant difference between the dominant and non-dominant sides when the leading foot lands (*P* = 0.59). However, when the trailing foot lands, there is a significant difference (*P* < 0.001), with the forefoot load on the dominant side being lower than that on the non-dominant side. For rearfoot load, there is no significant difference between the dominant and non-dominant sides when the leading foot lands (*P* = 0.59), but both proportions are less than 50%. When the trailing foot lands, there is a significant difference (*P* < 0.001) between the dominant and non-dominant sides, and both sides have rearfoot load proportions exceeding 50%.

**TABLE 4:** Comparison of plantar pressure distribution parameters between the dominant and non-dominant sides.

Items	Foot	Dominant side (M ± SD, %)	Non-dominant side (M ± SD, %)	t	*p*
Overall load	Leading foot	51.96 ± 6.20	58.16 ± 6.18	−7.42	<0.001
	Trailing foot	41.84 ± 6.18	48.04 ± 6.20	−7.42	<0.001
Forefoot load	Leading foot	55.54 ± 16.42	54.69 ± 14.63	0.54	0.59
	Trailing foot	40.45 ± 15.96	46.64 ± 15.47	−3.67	<0.001
Rearfoot load	Leading foot	44.46 ± 16.42	45.31 ± 14.63	−0.54	0.59
	Trailing foot	59.55 ± 15.96	53.36 ± 15.47	3.67	<0.001

#### 3.3.4 Comparison of plantar pressure distribution parameters between the leading foot and the trailing foot at different step heights

As shown in Table 5, when the dominant side serves as the leading foot, there is a significant difference between forefoot and rearfoot loads (*P* < 0.001), as when the dominant side serves as the trailing foot (*P* < 0.001). When the leading foot lands, the forefoot load is greater than the rearfoot load, while when the trailing foot lands, the forefoot load is lower than the rearfoot load. When the non-dominant side serves as the leading foot, there is a significant difference between forefoot and rearfoot loads (*P* < 0.001), as when the non-dominant side serves as the trailing foot (*P* = 0.019). When the leading foot lands, the forefoot load is greater than the rearfoot load, while when the trailing foot lands, the forefoot load is lower than the rearfoot load.

**TABLE 5:** Comparison of forefoot and rearfoot plantar pressure distribution parameters in the leading and trailing feet.

Location	Foot	Forefoot load (M ± SD, %)	Rearfoot load (M ± SD, %)	t	*p*
Dominant side	Leading foot	55.54 ± 16.42	44.46 ± 16.42	3.69	<0.001
	Trailing foot	40.45 ± 15.96	59.55 ± 15.96	−6.55	<0.001
Non-dominant side	Leading foot	54.69 ± 14.63	45.31 ± 14.63	3.52	<0.001
	Trailing foot	46.64 ± 15.47	53.36 ± 15.47	−2.38	0.019

## 4 Discussion

Varying step heights altered participants’ foot landing strategies, shifting from rearfoot to forefoot landing as the height increased. To be more specific, participants exhibited a preference for rearfoot landing regardless of whether the dominant or non-dominant side was used as the leading foot when descending 5 cm and 15 cm steps. However, at step heights of 25 cm and 35 cm, the preferred landing strategy shifted to forefoot landing.

The experimental results align with previous findings (Freedman and Kent, 1987) and support our research hypothesis. At the lowest step height of 5 cm, participants predominantly used a rearfoot landing strategy, while at the highest step height of 35 cm, they preferred forefoot contact. In normal gait, steps typically involve rearfoot contact, which results in minimal kinetic energy at lower step heights. As step height increases, forefoot landing becomes more prevalent, presumably to better absorb the kinetic energy acquired during the descent (van Dieën et al., 2008). A forefoot landing strategy is preferable for higher curbs or steps, as it allows for a more controlled descent and keeps kinetic energy within manageable limits. Therefore, a forefoot landing strategy is considered safer for descending steps than a rearfoot strategy, albeit at the potential cost of joint torque and gait speed efficiency (Buckley et al., 2008). Although a preference for rearfoot landing was noted at a 15 cm step height (56%/53%), landing strategies varied more at intermediate step heights. At a step height of 25 cm, 80%/86% of participants adopted a forefoot landing strategy, indicating a gradual shift in landing preferences with increasing step height. Previous research indicated that most landing strategies concentrated between step heights of 10 cm and 20 cm, with notable transitions in strategy from rearfoot to forefoot as height increases (5 cm = 96.36% rearfoot; 10 cm = 89.09% rearfoot; 20 cm = 78.18% forefoot)(Freedman and Kent, 1987). The height range of 17.8–22.5 cm, corresponding to some common step and curb heights (Axelson, 1999), coincides with the observed transition range in this study. This suggests these heights may be crucial for future research aimed at identifying mechanical factors influencing step descent in fallers and non-fallers. Specifically, most of the shifts in landing strategies were in the range of 10 cm–20 cm, whereas some of the common steps and curbs in real life are 17.8–22.5 cm in height, and these overlap in the range of 17.8–20 cm. Therefore, in the future, we need to avoid this overlapping height of steps and curbs in urban planning and building regulations as much as possible to prevent falls and sprains during the change of landing strategy.

Several factors may contribute to the preferred landing strategy at intermediate step heights, and one of them is related to individual’s height. Height differences, which determine leg length, allow individuals with shorter legs to transition from rearfoot to forefoot landing strategies at lower step heights. Indeed, in this experiment, for a 15 cm step height, the average height of participants who preferred a rearfoot landing strategy (1.78 ± 0.04 m) was greater than the average height of those who preferred a forefoot landing strategy (1.74 ± 0.07 m). Another potential factor influencing landing strategy is the strength of the lower limbs. Studies suggest that particularly quadriceps strength may be crucial in step descent (Gerstle et al., 2021). Although our study did not assess lower limb strength, all participants were healthy young males, excluding significant strength disparities due to lower limb diseases. Therefore, lower limb strength may not significantly influence the choice of landing strategy in this healthy young male cohort. Lastly, another influencing factor may be the approach speed (van Dieën and Pijnappels, 2009; Gerstle et al., 2017). One study found that as approach speed increased, the likelihood of a rearfoot landing strategy also increased (van Dieën and Pijnappels, 2009). However, all participants descended at their habitual speeds for transition step in this study (Lythgo et al., 2007; Rao et al., 2009; Buckley et al., 2010). Future studies could instruct participants to walk at specified speeds to better assess the impact of approach speed on landing strategy during single transition step descent.

In human bipedal motion, controlling dynamic stability is a key movement priority (AminiAghdam et al., 2019). During single transition step descent, potential errors exist, including stumbling or slipping during the loading phase or losing control of the center of mass (COM) during the descent phase (Templer, 1995). Observations of the body’s COM can be projected onto observations of the COP on the foot sole (Hak et al., 2013), showing the same trends (Vlutters et al., 2016). Consequently, COP parameters in the ML and AP directions, as well as the total adjustment velocity, 95% confidence circle area, ML range, and AP range increase with the increase in step height. This shift in the body’s COM leads to instability during the transitional landing phase. Descending steps requires more balance control than walking on level ground due to the lowering of the COM. Oates et al. (2005); Oates et al. (2017) observed that individuals tended to increase ML range to stabilize themselves during challenging walking scenarios. Additionally, rapid changes in COP during step descent may result from downward momentum being transferred to lateral momentum during the braking phase, indicating stronger braking forces. So our present study, from a kinematic perspective, reveals that an increase in step height leads to greater forward velocity of the COM, in response to an increase in AP-related plantar pressure center parameters (Vieira et al., 2017). This decrease in stability results from dynamic changes during step descent. While these studies explore the impact of body momentum on balance control at normal walking speeds, further research is needed to understand the relationship between whole-body COM, stepping patterns, and the influence of speed on dynamic posture control (AminiAghdam et al., 2019). Moreover, as step height increases, the fear of falling within the participants potentially intensifies, reducing dynamic balance ability and thus affecting the stability during subsequent transitions from higher step heights to level ground, which increases the risk of unstable landings or fall-related ankle sprains (Adkin et al., 2002; Patil et al., 2013; Cleworth et al., 2019). Heightened fear of falling due to greater heights results in excessive caution, affecting normal gait characteristics, muscle strength, and motor function (Hauer et al., 2009; Ayoubi et al., 2015), which significantly impacts daily life (Murphy et al., 2002; Franchignoni et al., 2005).

In the parameters related to the plantar pressure center, distinct biomechanical differences were observed in transitional step descent with different landing feet. Notably, COP parameters of the dominant and non-dominant sides in the ML and AP directions, as well as total adjustment velocity, 95% confidence circle area, ML range, and AP range, exhibit differences between the leading and trailing feet, with significant disparities in the AP direction and total velocity. This disparity may stem from variations in the movement patterns of the lower limbs. During the single support phase of forward descent, the trailing foot remains stationary, allowing time and space to position the leading foot (Pijnappels et al., 2005). Additionally, at the initial ground contact, the leading foot generates more momentum and AP positional displacement than the trailing foot (van Dieën et al., 2008). Individuals experience potential energy loss corresponding to step height during the process of descending steps. Part of this energy is absorbed by the trailing foot, converting it into kinetic energy. The leading foot must absorb this kinetic energy through eccentric contraction during landing, otherwise imbalance and falls may occur during the descent (van Dieën et al., 2007). According to the COP scatter plot, the mean Y of the leading foot exceeds that of the trailing foot. From the perspective of COP, the trailing foot shows fewer deviations and requires fewer postural adjustments than the leading foot. Indeed, van Dieën et al. (2007) suggest that a rapid response of the trailing limb is a reliable strategy for avoiding falls when unexpectedly encountering step descent in healthy young individuals. Regarding COP parameters in the AP and ML directions, a focus on coordinated control in the ML direction rather than forward progression is recommended. Research by Cui et al. (2020) indicated that stability in the ML direction is prioritized over the AP direction. Previous studies have also confirmed the crucial role of ML stability in movement processes (Krishnan et al., 2013; Eckardt and Rosenblatt, 2018).

Besides, interesting findings were observed regarding the vGRF; as step height increased, the vGRF decreased for the biped, leading and trailing feet measurements. This phenomenon could be attributed to changes in the landing strategy of the leading foot. Compared to rearfoot strike, forefoot landing exhibits smaller impact forces owing to increased plantar flexion at the ankle joint and the associated eccentric control by the calf muscles. As step height increases, the landing strategy shifts towards forefoot landing, resulting in prolonged support phase by the leading foot. During this phase, forward velocity significantly decreases, leading to a substantial reduction in the vGRF when both feet land (van Dieën et al., 2008). This observation aligns with the principle that higher approach velocities favor a rearfoot strike landing strategy (van Dieën and Pijnappels, 2009). This is consistent with the findings of van Dieën et al. (2008), who reported lower vGRF for individuals using a forefoot strike compared to a rearfoot strike. Additionally, the increased caution and slower movements observed at elevated step heights may reflect psychological factors such as fear and apprehension of falling, potentially explaining our findings (Patil et al., 2013). Finally, the study by van Dieën et al. (2008) suggested no significant differences in the dynamics of the trailing foot between the forefoot and rearfoot landing strategies with a descent height difference of 10 cm (van Dieën et al., 2008). Our experiment corroborated this conclusion and further revealed that significant differences in the vGRF of the trailing foot manifest only at step height differences of more than 20 cm.

The plantar pressure distribution data during the transition step, when the dominant side serves as the leading foot and the non-dominant side as the trailing foot, and when the non-dominant side serves as the leading foot and the dominant side as the trailing foot, consistently show that the overall load on the leading foot is higher than that on the trailing foot. However, the difference in overall load between leading foot and trailing foot of the dominant side as the leading foot and the non-dominant side as the trailing foot is slight. The reason why the overall load on the leading foot is higher than that on the trailing foot may be attributed to inherent gait differences between the leading and trailing feet during the entire single transition step descent, which consists of single support and double support phases (Vlutters et al., 2016). As the leading foot makes initial contact with the ground and the trailing foot swings, the leading foot bears the entire body mass, increasing load due to the prolonged single support phase. Secondly, as the non-dominant side may have slightly inferior adjustment and control capabilities compared to the dominant side, this potentially results in larger differences in load. Consistent with findings from the study by Cho et al., the leading foot tends to have a higher forefoot load and a lower rearfoot load (Cho et al., 2021), while the trailing foot exhibits opposite trends. Finally, this observation may be linked to differences in landing strategies between the leading and trailing feet. The landing strategy of the leading foot changes with variations in step height, while the trailing foot’s descent is more akin to a fixed swing. Consequently, the leading foot faces greater ground landing challenges than the trailing foot. Moreover, individuals exhibit varying pressure patterns and load control across different regions of the sole during dynamic postural stability (Rozema et al., 1996). This balance is primarily achieved by increasing the forefoot load on the leading foot (Cho et al., 2021) and the rearfoot load on the trailing foot.

Additionally, this study examined differences in overall load distribution between the dominant and non-dominant sides, revealing that the regulation of plantar pressure distribution on both feet differs between these sides during the balancing process. For the trailing foot, the forefoot load is lower and the rearfoot load is higher on the dominant side compared to the non-dominant side. Finally, for both the dominant and non-dominant sides, the leading foot consistently exhibits a forefoot load greater than the rearfoot load, with similar loads. In contrast, the trailing foot exhibits a lower forefoot load than the rearfoot load, but with larger differences, indicating that the trailing foot requires more rearfoot load for postural control on the dominant side. So, when the non-dominant side serves as the trailing foot, the distribution between the forefoot and rearfoot is more balanced, demonstrating superior posture control and balance capabilities. Studies indicate that changes in plantar pressure can lead to adverse outcomes, including excessive load on the metatarsal and heel regions, which potentially increase the risk of disease and fall-related injuries (Mickle et al., 2010; Rao and Carter, 2012). Therefore, using the dominant side as the leading foot and the non-dominant side as the trailing foot in single transition step descents results in a comparatively balanced load state, reflecting better postural control capabilities and low risk of injuries.

## 5 Limitations and future directions

As there are significant differences in gait, mobility, and psychology between men and women (Kerrigan et al., 1998; Gerstle et al., 2021), this study exclusively recruited healthy males to minimize gender-related variability in single step descent. Secondly, this experiment has not yet analyzed the changes in visual factors. Alterations in visual factors may affect the stability and balance of the body during descent at various step heights. Lastly, the experiment was conducted using participants’ preferred speeds for approach velocities. However, increased walking speeds may reduce stability upon foot landing (Süptitz et al., 2012; McCrum et al., 2019). Future research will explore the role of speed more thoroughly by having participants descend a single transition step at varied speeds, assessing its effects in this context.

## 6 Conclusion

Changes in step heights, landing foot, and footedness result in distinct foot landing strategies and plantar biomechanical characteristics during single transition step descent in healthy young males. As step height increases, plantar pressure center increases, while vGRF for the biped, leading and trailing feet decrease. The dominant side exhibited superior control ability to the non-dominant side, particularly when working as the leading foot. Together, as step height increased, participants tended to shift from a rearfoot to a forefoot landing strategy to absorb vertical reaction force, which may increase the risk of ankle sprain and falling, especially when the leading foot was non-dominant. Specifically, choosing the middle step height of 15 cm and 25 cm should not be too low (too much task volume) and too high (too much challenge and too much risk). Meanwhile, this paper suggests the use of the dominant side as the leading foot and the non-dominant side as the trailing foot as a single transition step through the steps of the program. It reflects a relatively balanced loading state and shows better human postural control and dynamic balance.

Observed shifts in plantar pressure and foot landing strategies, particularly with increasing step heights, suggest how the balance control and stability change in dynamic environments. These variations and adaptations may be critical in designing targeted interventions aimed at reducing fall risks across various populations. Variations in load distribution between the dominant and non-dominant sides underscore the role of lateralization in balance strategies, potentially informing personalized approaches in physical therapy and rehabilitation to address specific weaknesses or compensatory behaviors. These findings warrant further investigation into the neuromuscular and structural factors driving these differences, potentially guiding more tailored and effective fall prevention programs based on individual biomechanical profiles.

## Data Availability

The original contributions presented in the study are included in the article/Supplementary Material, further inquiries can be directed to the corresponding authors.
